# The Impact of Preterm Birth on Sleep through Infancy, Childhood and Adolescence and Its Implications

**DOI:** 10.3390/children9050626

**Published:** 2022-04-27

**Authors:** Jayne Trickett, Catherine Hill, Topun Austin, Samantha Johnson

**Affiliations:** 1 Department of Neuroscience, Psychology and Behaviour, University of Leicester, Leicester LE1 7RH, UK; 2 School of Clinical Experimental Sciences, Faculty of Medicine, University of Southampton, Southampton SO16 6YD, UK; c.m.hill@soton.ac.uk; 3 Department of Sleep Medicine, Southampton Children’s Hospital, Southampton SO17 1BJ, UK; 4 Neonatal Intensive Care Unit, Rosie Hospital, Cambridge University Hospitals NHS Foundation Trust, Cambridge Biomedical Campus, Cambridge CB2 0QQ, UK; ta338@cam.ac.uk; 5 Department of Health Sciences, University of Leicester, Leicester LE1 7RH, UK; sjj19@leicester.ac.uk

**Keywords:** sleep, preterm birth, infancy, childhood

## Abstract

There is emergent literature on the relationship between the development of sleep-wake cycles, sleep architecture, and sleep duration during the neonatal period on neurodevelopmental outcomes among children born preterm. There is also a growing literature on techniques to assess sleep staging in preterm neonates using either EEG methods or heart and respiration rate. Upon discharge from hospital, sleep in children born preterm has been assessed using parent report, actigraphy, and polysomnography. This review describes the ontogeny and measurement of sleep in the neonatal period, the current evidence on the impact of preterm birth on sleep both in the NICU and in childhood and adolescence, and the interaction between sleep, cognition, and social-emotional outcomes in this population.

## 1. Introduction

Preterm birth (<37 weeks of gestation) places children at increased risk for health and developmental problems compared with birth at term. Whilst severe sensory and motor disabilities affect a small proportion of survivors, the most common adverse outcomes are cognitive deficits and attention, social and emotional problems [1]. There is also increasing evidence that preterm-born children have poorer educational outcomes, including poorer academic attainment and an increased risk for special educational needs relative to term-born peers [2,3]. Current endeavours to identify targets for intervention have focused on elucidating the factors that mediate these outcomes. One factor that may contribute to the high prevalence of cognitive and academic difficulties in this population is poor sleep. Here we review the evidence relating to the impact of preterm birth on sleep throughout childhood and adolescence, including a review of sleep ontogeny and approaches to the assessment of sleep throughout childhood. We also discuss the association of sleep with cognitive and social-emotional problems and consider implications for the development of targeted interventions. 

## 2. Sleep during the Neonatal Period

### 2.1. Sleep Ontogeny during the Neonatal Period

Once the basic anatomical structures of the brain have developed early in pregnancy, there is a rapid and complex expansion of neuronal architecture which accelerates during the last trimester. This includes the growth of both thalamocortical connections, long- and short-range cortico-cortical connections [4], and the subsequent development of neuronal networks through synaptic pruning of these connections based on endogenous and exogenous activity. This period is also a critical phase in neuronal migration, oligodendrocyte precursor development, and the early development of functional networks. The preterm brain is susceptible to hypoxic-ischaemic injury, infection, and haemorrhage which can have profound effects on neuronal development; the preterm brain also develops in the relatively hostile environment of the NICU with loud noises and painful procedures all potentially affecting sleep [5,6].

The classification of different sleep states in foetal and neonatal life depends on a constellation of physiological, behavioural, and electrophysiological features [7]. In the foetus, sleep and the sleep-wake-cycle (SWC) are inferred from changes in behavioural states assessed using ultrasound examination. Early in gestation, foetal behaviour is observed as random movements; by 15 weeks, more coordinated patterns of motion develop [8] and, by 32 weeks, distinct clusters of behaviour can be observed based upon measurements of heart rate, body, and eye movements. These clusters are described as active sleep and quiet sleep with short periods of ‘wakefulness’. Active sleep is characterised by irregular heart rate and breathing movements, as well as the foetus having gross motor movements; conversely, quiet sleep is characterised by a regular heart rate and breathing movements and fewer body movements. Active sleep behaviour is more apparent than quiet sleep in the foetus with the latter appearing around 28–30 weeks [9]. The foetus cycles between active and quiet sleep in an ‘ultradian’ cycle of 70–90 min and there is some evidence that the transition time between these states may impact on later development of self-regulation [10]. As the foetus matures, the time spent in active sleep steadily decreases. Active sleep is implicated in processing sensory stimulation in the central nervous system [11,12], while quiet sleep is important for synaptic remodelling (Knoop et al., 2021 as cited in [13]).

Infants born preterm exhibit similar behavioural features to the foetus of an equivalent gestational age and the presence of rudimentary sleep states based on eye movements and EEG has been described in babies as young as 24 weeks of gestation [14]. EEG has been used extensively to study both brain maturation and the development of sleep and SWC in preterm and term-born infants [15]. While a 24-h circadian SWC does not develop until 3–6 months of age, preterm and term-born infants’ cycle between active/quiet sleep and wakefulness in short ultradian cycles. For example, cycling durations ranging from 37–100 min have been observed in preterm infants between 25 and 30 weeks of gestation [16]. By term, there is a more organised pattern of SWC with 3–4 periods of active/quiet sleep each lasting around 50–60 min followed by a period of wakefulness every 3–4 h [9].

The preterm infant spends up to 90% of its time asleep. Aside from active sleep, quiet sleep, and wakefulness, a fourth ‘indeterminate’ state has been described with features of both active sleep and quiet sleep, however, the time spent in this state decreases as the preterm infant matures and more discrete phases of sleep and wakefulness occur [17]. At 28 weeks’ gestational age, active sleep predominates then decreases steadily to account for around 50% of total sleep by term. In one study, the proportion of time spent in quiet sleep during the first week after birth in infants born between 27 and 30 weeks of gestation, without severe complications, increased to values similar to children born at full term prior to discharge at 36.9 to 38.1 weeks post-menstrual age [18]. The proportion of time spent in quiet sleep, and the average duration of time intervals spent in quiet sleep, predicted post-menstrual age with a reasonable degree of accuracy (R^2^ = 0.87) [18]. The maturation of sleep closely reflects cerebral maturation and so appears to follow a predictable pathway determined by gestational rather than chronological age.

Sleep is the primary activity of the developing brain in early life, so it is unsurprising that sleep plays a crucial role in brain development. The presence of SWC, sleep architecture and the percentage of 24 hour-time spent asleep during the neonatal period have all been associated with neurodevelopmental outcomes. For example, the absence of SWC assessed using amplitude-integrated EEG in the 4th week of life following birth at <30 weeks of gestation was highly prognostic of brain injury [19]. Using behavioural coding to identify the presence of Rapid Eye Movements (REMs) in active sleep, it was found that infants with severe brain injury who had a proportion of REM in active sleep above the median at 32–36 weeks’ post-menstrual age, had significantly higher cognitive test scores at six months of age compared with children with a lower proportion of REMs in active sleep. A high versus low proportion of REMs in active sleep was also predictive of cognitive test scores above birth weight, gestational age, medical risk, and maternal education [20]. In another study, a lower proportion of the night spent asleep based on actigraphy at 36 weeks post-menstrual age, indicating the maturity of sleep organisation, was associated with higher cognitive test scores at six months of age, explaining 25% of the variance [21]. The authors argue that decreased sleep duration reflects a more mature pattern of sleep, which is associated with better cognitive outcomes. However, as we discuss in the next section, actigraphy does not reliably assess sleep in infants born preterm [22].

While the development of sleep and SWCs coincide with the development of functional networks in the brain [9,12,23,24], establishing a causal link between sleep, functional connectivity, and early development remains elusive, not least because of the challenges of measuring sleep and functional connectivity both in utero and in preterm infants in the neonatal unit.

### 2.2. Methodological Approaches to Assessing Sleep in Preterm Neonates

Despite the fact that preterm infants spend most of their time asleep and the importance of sleep for brain development, it is perhaps surprising that sleep is not routinely monitored during the neonatal period. ‘Sleep studies’ in the neonatal unit tend to be focused primarily on infants with chronic lung disease and evaluation of obstructive and central sleep apnoea which can lead to apparent life-threatening events, rather than measuring the quality of sleep in relation to neurodevelopmental outcome [23]. Whilst sleep duration, quality, and architecture in childhood can be studied using electroencephalography (EEG) or actigraphy (see Section 3), in preterm neonates actigraphy has poor specificity in differentiating wake from sleep [22] and EEG alone cannot reliably differentiate between active and quiet sleep prior to 30 weeks of gestation [24]. Therefore, the development of sleep for infants born extremely preterm, i.e., from 24 weeks of gestation, cannot be reliably studied using EEG or actigraphy in the first few weeks ex-utero. However, a recent observational checklist has been developed to identify states of active sleep, quiet sleep, and wake in infants assessed 25 + 0 to 36 + 6 weeks at post-menstrual age without significant brain injury, congenital malformations, seizures and not receiving invasive respiratory support, with very good inter and intra-rater reliability [25]. This checklist included observation of eye movements, body movements, facial movements, vocalisations, heart rate, respiratory patterns, and activity levels. 

In recent years there has been growing interest in sleep monitoring coinciding with developments in remote monitoring technologies and approaches to automated data analysis. The ultimate goal would be a non-contact system that could assess the different sleep states as well as periods of wakefulness. A cot-side display could inform the medical and nursing staff of the infant’s sleep/wake state enabling care, feeding, and other procedures to be performed in transitional or wake states to protect natural sleep cycling. A number of reduced/non-contact measurement systems have been proposed [26]. Some technologies, such as Ballistocardiography (BCG), which measures heart rate (HR) and respiratory rate (RR) by way of mechanical deformation of sensors, usually placed in a mattress, are commercially available for use in older infants–primarily to detect apparent life-threatening events rather than sleep patterns. Others, such as Impulse-Radio Ultrawideband Radar (IR-UWB) or photoplethysmography imaging remain very much experimental tools at this stage [27,28]. Further research is needed to validate these measurement tools in infants with a post-menstrual age <32 weeks. 

### 2.3. Impact of NICU Environment on Sleep in Preterm Infants

The development of sleep should occur in the protected environment of the womb. Despite the best efforts of nursing teams, the neonatal unit can be considered a hostile environment, particularly with regard to sleep hygiene. As well as intrinsic pathologies associated with prematurity, such as cardiorespiratory instability, hypoxia-ischaemia, intraventricular haemorrhage, sepsis, etc., the extrinsic environment provides an inconsistent and hostile sensory environment for the development of sleep. 

The preterm infant is exposed to significant background ‘white noise’ from machineries such as ventilators or other forms of respiratory support, sudden noises due to alarms going off, or incubator doors being closed inappropriately, as well as the background noise of both parents’ and healthcare professionals’ voices. Exposure to loud, sudden, or sustained levels of noise creates excessive auditory stimulation, resulting in both sleep disturbance and changes in systemic physiology [29]. Unfortunately, there is a paucity of evidence regarding the impact of noise on sleep in critically ill neonates. Kuhn and colleagues, in an observational study of 26 preterm infants between 26–31 weeks of gestation showed that even moderate noise between 5–15 dB above ambient background levels could interrupt sleep patterns. They went on to show that infants’ sleep was interrupted on average 18 times a day and that over a nine-week hospital stay this could equate to 1134 disturbances to sleep solely due to noise [6].

The preterm infant is also exposed to numerous tactile stimuli, many of which are painful or noxious. Few studies have directly studied the impact of painful stimuli on sleep architecture in preterm infants. Axelin and colleagues undertook a prospective randomized placebo-controlled cross-over trial to investigate the effect of pain management on sleep in preterm infants. Interestingly this study did not show an effect of non-pharmacological pain management on sleep structure; however, the use of opioids led to a significant reduction in the amount of REM (active sleep) [30]. Mechanical ventilatory support in preterm infants has been associated with delayed maturation of sleep at term and, given the judicious use of opioids in ventilated preterm infants, the combined effect of mechanical ventilation and opioid administration is a potential cause of concern and warrants further investigation [31].

### 2.4. Summary: Sleep during the Neonatal Period

Optimising healthy sleep in infants cared for in the neonatal unit is of primary importance given the association between sleep organisation and neurodevelopmental outcomes. Further research is needed to validate unobtrusive methods for long-term monitoring of sleep in preterm infants, to identify the developmental course of sleep maturation and individual differences that may be associated with neonatal pathologies in preterm populations. 

## 3. Sleep during Childhood and Adolescence

As preterm-born babies are discharged home from neonatal care, the focus of sleep assessment shifts to the ecological assessment of sleep in the home environment, often including parents’ perceptions of whether their child’s sleep is a problem or not. This section describes subjective and objective methods that can be used to quantify sleep and assess sleep-disordered breathing (SDB). These methods are evaluated in the context of their use in the follow-up of children born preterm to explore the role of sleep on cognition, behaviour, and mental health in this population. 

### 3.1. Assessing Sleep and Its Disorders in Childhood and Adolescence 

**Figure 1:**
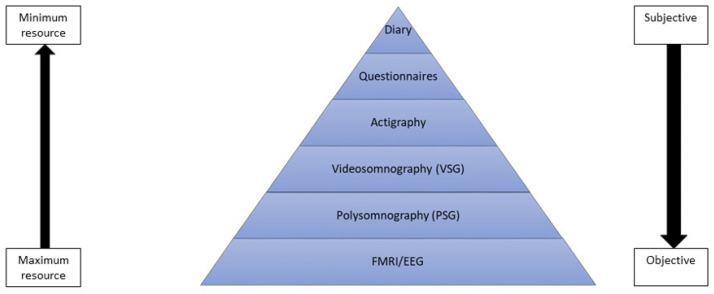
Trade-off between cost/availability and sensitivity of measures of sleep.

Harnessing meaningful data on sleep and its disorders in children and adolescents is key to understanding outcomes and cognitive correlates. A variety of technologies are available that differ in the dimensions of sleep captured, ease of collection, ecological validity, and reliability. They range from cheap, accessible, subjective self-report measures which can easily be included in a questionnaire battery in a follow-up wave of data collection in a large cohort study to laboratory ‘gold standard’ polysomnography (PSG) for children with suspected SDB (see Figure 1). 

#### 3.1.1. Objective Measures of Sleep

Quantity (e.g., total sleep time, both night and 24-h) and quality of sleep (sleep efficiency or longest sleep period) can be assessed using either actigraphy or polysomnography. Actigraphs (typically worn on the non-dominant wrist or ankle) measure activity levels continually in three plains (tri-axial accelerometry). Software algorithms interpret sleep-wake patterns based on a drop-in movement associated with sleep. They offer an objective, non-invasive, relatively inexpensive (compared to PSG) method to record multiple nights of sleep data in the home. The use of actigraphy, both in clinical practice and research, has increased exponentially over the past decades [32]. The American Academy of Sleep Medicine (AASM) recommends actigraphy as an appropriate assessment tool in clinical paediatric practice for the assessment of insomnia, and circadian rhythm disorder and in preparation for multiple sleep latency testing. A minimum of seven days of recording is recommended [33] to ensure five days of usable data. For weekday/weekend differences two weeks are recommended. Technical specifications of devices, and in turn their sensitivity to detect sleep, vary considerably and few have been formally validated against PSG in children. In general, sensitivity to detect sleep is high but specificity to detect wake is low with some developmental differences (see Table 1). There are no international guidelines on technical specifications for recordings or analysis and subsequently no normative data in children. Meltzer (2012) recommended a standard checklist for reporting the use of actigraphy in children, e.g., technical specification of device and software, number of days of recording (free sleep versus scheduled sleep) to standardise future research [34]. Actigraphy is the only objective assessment method that facilitates the measurement of variability in sleep quantity and quality; both short-term night-night variability/weekday to weekend variability, as well as long-term changes in sleep across childhood. Given the association between variation in night-to-night sleep and behaviour and emotional symptoms in children and adolescents in the general population [35], actigraphy, which can measure sleep over a number of days, would be best suited to research exploring the association between sleep and emotional and behavioural problems in children born preterm. 

One birth cohort reported a reasonable completion rate of 66% for all participants enrolled in a follow-up wave of data collection which analysed actigraphy data from one 24 h period in 12-month-old infants where an actigraph was worn continuously for three days [36]. However, actigraphy should ideally be paired with sleep diary data to identify artefacts and to accurately assess sleep timing and sleep onset latency from parent-reported bedtime and morning wake time. Actigraphs can be sent and returned by courier to participating families and therefore have the potential to be used in large-scale studies.

In addition to providing a gold-standard assessment of sleep quantity and quality, polysomnography facilitates sleep staging scoring based on scalp electroencephalogram (EEG) electrodes, alongside changes in muscle tone and eye movements measured by sub-mental electromyogram (EMG) and electro-oculogram (EOG) respectively. Visual inspection of EEG/EOG and EMG signals in 30s epochs identifies changes in spectral EEG power and characteristic EEG features such as sleep spindles to categorisation of sleep into rapid eye movement (stage R) and non-REM sleep stages (N1–N3) which occurs in predictable cycles. 

Obstructive sleep apnoea (OSA) can only be reliably quantified by polysomnography [37] which is resource-intensive. SDB describes a spectrum of breathing abnormalities in sleep from a partial reduction of airflow measured at the nose (hypopnoea) to complete cessation of airflow (apnoea). Hypopnoea and apnoea may be caused by upper airway obstruction (characterized by continued respiratory effort) or loss of central respiratory drive (absence of respiratory effort). Sleep-related hypoventilation in infants and children can be caused by derangement in central ventilatory control, mechanical impairment (i.e., OSDB, chest wall abnormalities, obesity hypoventilation syndrome), and abnormalities in gas exchange leading to increased dead space ventilation (i.e., chronic lung disease). OSA is the most prevalent form of SDB in childhood. The thresholds for defining how many obstructive hypopnoeic and apnoeic events per hour are required for a diagnosis of OSA vary between studies. A recent European consensus group has categorized OSA in children aged 1–23 months as mild: obstructive AHI 1–5 episodes/hour; moderate: obstructive AHI > 5–10 episodes/hour and severe: obstructive AHI > 10 episodes/hour [38].

The choice of technology is important and device settings, duration of recording, and analysis software are crucial to interpreting meaningful results [39]. PSG is the only widely available tool for research where sleep macrostructure (i.e., sleep architecture/sleep stage composition) or microstructure (e.g., sleep spindles) are variables of interest. For example, ambulatory PSG has demonstrated a relationship between memory consolidation and regional fast spindle density and between the slow spindle and general cognitive abilities in children and adolescents [40]. PSG is resource-intensive, traditionally performed in a sleep laboratory attended and scored by specialist staff. Recent technical advances including small, lightweight, portable equipment make low-cost home studies feasible. In clinical settings better subjective sleep quality and high rates of technical success (87%) have been reported [41] than in ambulatory settings although children’s tolerance and need for lead replacement means its use is limited to only one or two nights. In experienced hands, home PSG offers an attractive alternative to in-lab studies for research where sleep macrostructure or microstructure is of interest. However, home-based PSG studies still require a technician to set up the study and analysis is resource-intensive so this approach has its limitations for large cohort studies. In practice, where PSG is not feasible for fiscal or practical reasons, proxy measures can be used. If a full PSG study cannot be conducted, pulse oximetry provides data on oxygen saturation which captures nocturnal hypoxia, an important but not unique cause of OSA-related cognitive impairment. 

#### 3.1.2. Subjective Measures of Sleep

Sleep questionnaires provide a simple accessible means of assessing dimensions of sleep and can be used across large samples. Over 200 questionnaires have been developed to assess various aspects of sleep and its disorders in children. For a comprehensive review see [42,43]. The use of sleep questionnaires facilitates the assessment of sleep in relation to behavioural, emotional, and social outcomes, which are typically assessed via parent questionnaires in cohort studies. 

For general sleep problem screening, two parent-report questionnaires are commonly used in the paediatric literature: the ‘Child Sleep Habits Questionnaire’ [44] and the ‘Sleep Disorders Scale for Children’ [45]. The latter has undergone the most robust development and validation process and more recent studies have demonstrated its excellent fit with sleep disorder classification as well as utility in clinical populations with developmental disorders [42]. Australian researchers addressed the limitations of these two questionnaires and generated a new 26-item ‘Omnibus Sleep Problem’ questionnaire for children which combines aspects of both the Child Sleep Habits Questionnaire and the Sleep Disorders Scale for Children questionnaire [46]. While this has not been extensively tested in different populations, it appears a promising new tool for children aged 5–10 years. For longitudinal studies that include pre-school measurement points, developmental continuity is a problem that has not been resolved by current questionnaires. The best tool for assessing sleep in pre-school children remains the ‘Brief Infant Sleep Questionnaire’ [47] which provides an assessment of sleep patterns, sleep ecology, and parental perceptions of sleep suited for infants and toddlers 6–36 months. The original scale has been revised (BISQ-R) to include a wider array of sleep behaviours and outcomes with normative reference values recently develop in a US population [48]. 

There are many parent-report questionnaires designed to screen for specific sleep disorders. Given the cost associated with PSG assessment for OSA, a reliable screening tool is appealing. The most widely used tool is the ‘Pediatric Sleep Questionnaire’ (PSQ) [49] with reported sensitivity between 0.81 and 0.85 and specificity of 0.87 to detect an obstructive apnoea hypopnoea index (OAHI) of ≥5/hour. This has been validated in many international samples [42] although the presence of a behavioural sub-scale limits its application to children with developmental disorders associated with behavioural co-morbidity. While the gold standard reference for validation of such questionnaires is the OAHI, this measure reflects only one night in a child’s development. For researchers interested in how longitudinal exposure of the brain to OSA-related insult impacts cognitive outcomes, a parent report measure that reflects longer time frames may be of more value. In support of this, a recent study noted that parent reports of snoring better predicted cognitive outcomes than polysomnography-derived OAHI [50]. 

### 3.2. Sleep in Childhood and Adolescence following Preterm Birth

#### 3.2.1. Sleep during Infancy and the Pre-School Period

Evidence for the impact of preterm birth on sleep beyond the neonatal period is mixed. A summary of studies assessing sleep in preterm infants following discharge from the hospital and in preschool-aged children is presented in Table 2. 

In one of the earliest studies, using parental observation over 14 days every month until 12 months corrected age, infants born <37 weeks of gestation without neonatal morbidities had delayed development of sleep-wake periodicity when referenced to post-natal weeks, but not post-conceptional weeks. There was no significant difference in daytime or nocturnal sleep duration, nor in the longest uninterrupted sleep period between infants born preterm and at term [60]. 

In seven of the ten studies that assessed nocturnal sleep duration, there was no difference between preterm and term-born infants and toddlers (Table 2). In one study [58], parents’ estimated sleep duration was longer in preterm (<37 weeks of gestation) infants, although the frequency of night awakenings was greater in preterm compared with term-born children. In the two studies which recruited very preterm children (≤32 weeks of gestation), there was mixed evidence of both greater duration [55], no difference in duration [52], and no difference in frequency [52] or lower frequency [55] of night wakings and a higher nocturnal activity index according to PSG [52] in very preterm compared with term-born children. The limited number of studies of children born very preterm combined with the exclusion criteria of participants with severe impairments does not enable the generalisation of these findings to children born <32 weeks of gestation. In addition, only two studies assessed sleep using actigraphy and only in 14 and 21 infants born preterm. 

Despite the limited evidence for sleep disturbance in children born preterm, when parents were asked about their perception of their child’s sleep, in three out of the four studies where these data were obtained, a greater proportion of parents of preterm than term-born children considered their child to have a sleep problem or were concerned about their child’s sleep [52,53,59] but this did not differ between the group born <37 weeks of gestation and the preterm group in one study [51]. This finding is particularly important when considering that sleep efficiency and children’s longest sleep duration accounted for 71% of maternal parenting stress in mothers of preterm-born infants at 10–22 months corrected age [53]. One potential confounding factor in the study of sleep in the preterm population could be that parents of children born preterm, who may have had significant concerns over their child’s health whilst on the neonatal unit, may monitor their child’s health and behaviour more closely than parents of children born at term [64]. Therefore, whilst acknowledging parents’ concerns about any perceived sleep problems, the use of objective assessment in conjunction with parent-report would be beneficial.

Sleep problems are common in the general population but at age 4–6 years, sleep problems were most frequent among children born very and moderately preterm compared to those born late-term. In another study, children born <32 weeks of gestation aged 3–6 years (*n* = 146) had significantly higher scores on the initiating and maintaining sleep, sleep-disordered breathing, and sleep hyperhidrosis subscales than the control group children, but not on the parasomnias, disorders of excessive somnolence and nonrestorative sleep subscales of the ‘Sleep Disturbance Scale for Children’ [62]. However, of note, the control group for this study was comprised of children without developmental, mental, or physical disabilities or long-term prescribed medication rather than children born at term.

In summary, studies that included infants or pre-school children born <32 weeks of gestation do not provide a representative overview of sleep in this population due to the small sample sizes [52,55] and the lack of a term-born control group in the largest study [62] (see Table 2). For the broader population of all preterm-born children <37 weeks of gestation, there is some evidence of parents having concerns about their child’s sleep or reporting scores that indicated a non-specific sleep problem [52,53,56,59,63]. Four studies indicated no difference in night wakings for children born <37 weeks of gestation compared with controls [54,55,57,60], four studies indicated greater night waking for the preterm groups [52,53,59,61] and one study indicated no significant differences or a smaller proportion of parents reported night waking problems in the preterm group [61]. Further research is needed with larger, representative samples and the use of objective assessment to evaluate sleep and selection of appropriate control groups in infants and preschool children born preterm. 

#### 3.2.2. Sleep during Middle Childhood and Adolescence

A recent review provides a synthesis of nine studies on sleep in children born preterm from ages five to 18 years [65]. Seven of these nine studies used PSG or EEG to assess sleep quantitatively. The others used either an un-validated questionnaire or an interview schedule. When accounting for the quality of the studies, there was strong evidence for increased night waking and moderate evidence for earlier bedtimes in children born preterm compared with those born at term. The evidence for differences in sleep architecture however was mixed. Across the studies included, there was no significant difference in sleep duration using objective measures, with sleep duration mainly in accordance with AASM recommendations [65,66]. 

Since the publication of this review, two further studies using validated questionnaires have compared sleep disturbance between children born <27 weeks of gestation at age 11 years [67] (*n* preterm = 165) and children born <32 weeks of gestation aged 5–9 years [68] (*n* preterm = 102) relative to children born at term. Both studies showed that relative to controls, the preterm group had greater parent-reported SDB or snoring. In Brockmann and colleagues’ study, very preterm-born children had higher total scores, indicating more problematic sleep, higher scores on the sleep-wake transition, sleep-breathing disorders, and sleep hyperhidrosis subscales of the ‘Sleep Disturbance Scale for Children’ [68]. There was no significant difference in the disorders of initiating and maintaining sleep, disorders of arousal, and disorders of excessive somnolence subscales. In contrast, the sleep onset delay, daytime sleepiness, and night waking subscales of the ‘Children’s Sleep Habits’ questionnaire were higher for the extremely preterm group in Trickett and colleagues’ (2021) study, with no difference in the sleep duration subscale score between the preterm and term-born groups [67]. Trickett and colleagues’ (2021) study had no exclusion criteria, whilst Brockmann and colleagues (2020) excluded children with current chronic diseases and prescribed medication. 

Another recent study assessed sleep quality and quantity and macro- and micro-architecture in children born <37 weeks of gestation with SDB but without developmental disabilities [69]. This study found no significant differences in sleep quantity, quality, or macro-structure between the preterm and term-born groups who were matched on the severity of SDB [69]. Whilst this study suggests that SDB does not differentially impact the sleep of children born preterm compared with children born at term at a macro-level, at a micro-level, there was greater slow-wave activity in the F4 and C4 areas in the second non-REM period of the night in the preterm group. The authors suggested that the finding demonstrates an accumulation and reduced dissipation of sleep debt [69]. As discussed in Section 4, more research is needed to identify whether sleep disturbance/SDB differentially affects children born preterm, who have lower neuropsychological capacities [70] compared with children born at term without any neuropsychological impairments. A key limitation of much of the existing literature is the exclusion of children born preterm with neurodevelopmental disabilities or with health conditions. The use of a representative sample of children born at the earliest gestations is important, given the increased prevalence of sleep problems among children born <28 weeks of gestation with an increasing degree of disability [71].

Despite the majority of studies having used PSG or questionnaires to assess sleep in childhood following preterm birth, only two studies used actigraphy to assess sleep quality and duration among children or adolescents born preterm [72,73]. Whilst variability of sleep duration, sleep onset latency, and bedtimes across the 14-day assessment period were reported for the 188 children with a birthweight of 500–1250 g aged 5–12 years in Biggs and colleagues’ (2016) study, the duration of monitoring being a key benefit to the use of actigraphy, the lack of a comparison group hindered the interpretation of these findings [72]. Children in this study slept for one hour less than recommendations for this age group [72]. However, an actigraphy study of 146 adolescents aged 16–19 years born at <37 weeks of gestation found no significant differences in weekend and weekday sleep duration between adolescents born preterm and at term, although adolescents born preterm did have significantly earlier weekend and weeknight bedtimes and weekend wake times than their term-born peers. In this study, adolescents born preterm had fewer nocturnal arousals and reported less daytime sleepiness than term-born adolescents [73]. The onset of puberty affects the circadian timing of sleep with evening chronotype preference peaking at 16 and 17 years for girls and boys respectively [74]. As the proportions of children born <26 weeks of gestation who have entered puberty do not differ compared with children born at term at 11 years [75], it is unlikely that these differences in circadian timing can be attributed to delay in pubertal development. Further research is needed to understand these findings which may also reflect social and environmental factors. 

#### 3.2.3. Sleep-Disordered Breathing in Childhood following Preterm Birth

Sleep duration and quality are not only impacted by the child’s environment and learned behaviours but also by defined sleep disorders. Preterm birth is a well-established risk factor for SDB in childhood [38,74,75] and early adulthood [76]. The unadjusted odds ratios range from 5.0 (1.6 to 20.1) for children born <36 weeks of gestation at ages 8–11 when OSA was defined as an obstructive apnoea hypopnoea index ≥ 5 events per hour [77] to 6.7 (2.3 to 19.5) using the definition of parent-reported habitual snoring (snoring more than half the time) for children born <27 weeks of gestation at age 11 [67]. 

Risk factors for OSA (≥2 apnoea-hypopnea events per hour and/or a history of adenoidectomy/tonsillectomy) in children born with a birthweight of 500–1250 g include chorioamnionitis and multiple births, whilst protective factors include white maternal ethnicity and older maternal age [78]. Decreasing gestational age conferred an additional risk for OSA (≥5 events per hour) in children aged 2–22 months following preterm birth but not clinical morbidity (e.g., intraventricular haemorrhage, necrotizing enterocolitis) [79]. Although in another study, neither gestational age nor neonatal morbidity was associated with SDB (obstructive apnoea index ≥ 1 or AHI ≥ 5) at ages 8–11 years in children born <37 weeks of gestation [80]. Having a narrow hard palate at six months was associated with an increased risk for SDB among preterm-born infants [58]. 

Recommendations for assessing SDB in preterm infants have been published by Joosten and colleagues (2017) [23]. With reference to childhood, the National Institute of Health and Care Excellence guidelines for the developmental follow up of children and young people born preterm in the UK recommend that clinicians should check for symptoms of sleep problems, including SDB, at two and four years of age for children born <30 weeks of gestation and <28 weeks of gestation, respectively [81]. Identifying and treating SDB in children born preterm is particularly important, as SDB may confer additional neurocognitive risk in this population. For example, a greater fall in cerebral oxygenation whilst asleep was found in preterm-born infants at 2–4 weeks, 2–3 months, and at 5–6 months of age compared with children born at term [82]. Among children with SDB, those born preterm did not differ in respiratory parameters, sleep quality, duration, or sleep macro-architecture parameters from children born at term, but preterm children had a greater slow-wave and theta activity in non-REM stage 2 sleep, indicating increased sleep debt [69]. In addition to these differences in neurological activity during sleep, there is some evidence for greater impairments in cognitive and academic measures in preterm than term-born children with SDB. Habitual snoring/SDB was associated with poorer simultaneous information processing, academic achievement, and verbal knowledge in children born preterm with a low birthweight aged 8–11 years, but not in term born-children [83]. Whilst the mechanism for poorer cognitive outcomes in children with SDB, hypothesized to be attributed to either hypoxia or daytime sleepiness attributed to fragmented sleep, is unclear, the evidence for cognitive impairments in children born preterm with snoring indicates that a broad definition of SDB should be used in further research in preterm populations. It is worth noting that the trajectory of SDB has not been studied in children born preterm. The early onset of snoring is hypothesized to have greater neurodevelopmental implications than late-onset snoring [84]. Further research on the impact of snoring on cognition is needed with children born preterm who have lower global and specific cognitive impairments relative to children born at term [70]. This rationale is evidenced from research in the general population, in which school-aged children with SDB have poorer scores on global cognitive assessments, such as IQ [85] not on standardised assessments of working memory, inhibition, shifting, or vigilance [86] compared with children without SDB. However, these scores tend to remain within the average range [85].

### 3.3. Summary: Sleep and Sleep Disordered Breathing in Children Born Preterm

In summary, there is emerging evidence of an excess of night waking and earlier bedtimes among children born preterm compared with those born at term from studies using questionnaires and PSG. However, more research is needed using actigraphy to assess whether sleep quality and duration vary to a greater extent among children born preterm. In addition, future research needs to include age and sex-matched term-born control group and a representative sample of children born very preterm to enable the interpretation of actigraphy data given the lack of paediatric norms. Preterm birth is an established risk factor for SDB. Further research is needed on the daytime impact of sleep disturbance and snoring, in this population; the extant literature is discussed in Section 4.

## 4. Sleep and Cognitive and Socio-Emotional Problems in Children Born Very Preterm

Children born very preterm are at increased risk for inattention, social difficulties, and anxiety compared with children born at term [1]. It is possible that sleep disturbance may exacerbate symptoms of this ‘preterm behavioural phenotype’. Indeed, sleep is implicated in social, emotional, and attentional outcomes for toddlers born preterm. Children born <32 weeks of gestation aged 12–31 months who had lower social and emotional competence had shorter nocturnal and total sleep duration compared with children born preterm with social and emotional competence scores in the average range [55]. At age two, children born <37 weeks of gestation with higher parent-rated sleep difficulties scores had significantly poorer parent-rated attention (r = −0.32) [56]. In toddlers born <36 weeks of gestation and birthweight < 1500 g, at age 1.5 years, greater variation in wake-up time over seven days was associated with lower cognitive test scores [87]. In a longitudinal study with families of children born <37 weeks of gestation, there was an association between a greater number and duration of parent-reported nocturnal awakenings with atypical motor outcomes assessed on the Denver-II and the Alberta Infant Motor Scale at six months corrected age, but at 12 months corrected age, no such relationships were found [88]. However, in this study, it was not clear how atypical development was defined nor the rationale for exploring the development of motor skills and sleep.

Studies of the association between sleep, cognitive and social-emotional outcomes in other clinical populations may shed light on these associations in preterm-born children. Preschool children with autism have greater intra-individual variation in bedtime, sleep efficiency, and sleep duration, i.e., in the night-to-night variation compared with typically developing children [89]. There are mixed findings for children with ADHD. Whilst no difference in variation in sleep quality and duration between primary school-aged typically developing children and children with ADHD have been observed, although this study may have been underpowered [90], a larger study found that adolescents with ADHD had greater variability in actigraphy-assessed sleep timing and quality compared with adolescents without ADHD [91]. In a study of sub-clinical inattention symptoms in a sample of adults from the general population, higher inattention scores were associated with more variable reaction times on an inhibition task, but only following sleep deprivation [92]. This is suggestive of increased vulnerability to poorer cognitive performance following sleep deprivation when trait-inattention is higher. Given the increased risk for ADHD symptoms and autistic traits in individuals born extremely preterm and the association between variation in night-to-night sleep and behaviour and emotional symptoms in the child and adolescent general population [35], and the emerging research on the risk of sleep disturbance in childhood following preterm birth, research on the impact of sleep deprivation and the variability of sleep timing and quality on cognitive functioning in the preterm population is imperative. 

Studies have shown that sleep problems and poorer sleep efficiency are associated with poorer executive function, selective attention, mathematical ability, and visuospatial memory among children born very preterm but not at term [93,94]. However, there was no interaction between the number of nocturnal wakings, duration of slow-wave sleep, and REM sleep and preterm birth status on dual motor and cognitive performance tasks according to prematurity status in another study [95]. Differences in sleep micro-architecture between children born preterm and at term may reflect the resources drawn on to manage increased cognitive effort due to executive function deficits, as increased slow-wave activity in the frontal cluster was associated with executive function performance more strongly in the preterm compared with the term-born group [96].

With reference to emotional and behavioural symptoms in childhood, children born <28 weeks of gestation or with a birthweight < 1000 g with parent-reported difficulty falling asleep or frequent awakenings had higher parent-reported emotional, hyperactivity/inattention, conduct, and peer problems compared with preterm-born children without these sleep problems [97]. Parent-reported night waking also mediated the relationship between extremely preterm birth and ADHD and emotional symptoms [67]. These studies provide preliminary evidence for the association between parent-reported sleep disturbance and aspects of the preterm behavioural phenotype, but further research is needed with quantitatively assessed sleep, including night-to-night variation assessed using actigraphy. This would also mitigate against any reporting bias when relying on parent-report for both sleep as the predictor variable and socio-emotional problems as the outcome variable. A brief intervention delivered by practitioners which included using sleep hygiene and behavioural strategies for children with ADHD and co-occurring sleep problems was effective in reducing ADHD symptoms (moderate effect size) and large effect sizes for reducing sleep problems after three months [98]. Similarly, cognitive behavioural therapy and a behavioural intervention targeting anxiety and sleep problems in children with generalised anxiety disorder show moderate to large effect sizes for reducing parent-reported anxiety and parent- and child-reported sleep problems [99]. Therefore, interventions targeting sleep disturbance could be used to improve behavioural and emotional symptoms in addition to sleep in children born preterm.

## 5. Conclusions

Further research is needed to identify and validate techniques to assess sleep in neonates born at the most preterm gestations and to better understand the relationship between sleep and early brain development. Objective methods to assess sleep in childhood are well-validated across clinical and general population samples. Combined with the emerging evidence of the impact of sleep and sleep-disordered breathing on cognition/behaviour following preterm birth, further research is needed in a large, representative sample of children born very preterm to assess sleep. To assess sleep, robust objective measurements such as actigraphy studies are needed. The relationship between sleep quality, duration, and variability with comprehensive assessments of attention, working memory and visuospatial skills, alongside emotional and behavioural symptoms, could then be studied. Should impaired sleep quality, shorter sleep duration, or snoring be associated with poorer cognitive, emotional, or behavioural outcomes, a psychoeducational intervention and/or the appropriate treatment of co-morbid sleep disorders could be implemented to support families with their child’s sleep with a potential transfer to other cognitive and behavioural outcomes. 

## Figures and Tables

**Table 1:** Age-specific sensitivity and specificity of actigraphy to detect sleep.

Age Range	Sensitivity Range	Specificity Range
Infants	83.4–99.3	17.0–97.8
Toddler	87.7	76.9
Pre-schoolers	97.0	24.0
Adolescents	95.0	74.5
Multiple ages	82.2–90.1	50.9–72.8

Reprinted from Use of actigraphy for assessment in paediatric sleep research, 16, Copyright (2012), with permission from Elsevier [34].

**Table 2:** Sleep following preterm birth in infancy and the pre-school period.

					Findings Relative to Term-Born Control Group							
Authors	Method of Assessment of Sleep	Gestational Age	Age at Assessment	N Born Preterm	Night Waking	Sleep Duration Night	Sleep Duration Day	Sleep Timing	Sleep Onset Latency	Parent Perception of Sleep Problem	Other	Study Limitations
Sleep following preterm birth in infancy/toddlerhood following discharge from hospital												
(Akkus and Bahtiyar-Saygan, 2022) [51]	Parent report: Brief Infant Sleep Questionnaire	<37 weeks of gestation without significant neurodevelopmental problems or severe medical conditions	6–17 months corrected age	40	X both in number of night wakings and duration of nocturnal wakefulness	↓	x	Significantly later bedtime in the PT group		x	No significant group difference in the percentage of children who were classified as a poor sleeper (waking >3 times per night, spending more than one hour awake at night, or spending less than 9 h asleep). No significant group difference in bedtime difficulty score	Small sample size, relied on parent-report. Sample not representative of children born <37 weeks with significant neurodevelopmental disorders or medical conditions
(Asaka and Takada, 2010) [52]	Actigraphy	<1500 BW and <32 weeks of gestation without severe illness or congenital abnormality or use of medication with sedative effects	12 months corrected age	14	X both number of wakings and duration of nocturnal wakefulness Higher night-time activity index in PT group	↓	x	Earlier bedtimes and wake time in PT group		Greater proportion of parents concerned about child’s sleep in PT group	No significant difference in longest sleep episode or sleep efficiency	Small sample size. Sample not representative of children born <32 weeks with neontal morbidities
(Asaka and Takada, 2013) [53]	Actigraphy	<36 weeks of gestation and <2500 BW. No neurological or developmental problems	10–22 months corrected age	21	X both number of wakings (over 5 min) and duration of nocturnal wakefulness	↓ in PT group for infants ≤14 months of age X between the groups at over 15 months of age	↑ infants over 15 months in PT group	x		Greater proportion of parents concerned about child’s sleep in PT group	Sleep efficiency and longest sleep episode (whilst both did not significantly differ between the two groups) significantly accounted for 71% of maternal parenting stress	Small sample size. Sample not representative of children born <36 weeks with developmental problems
(Blair et al., 2012) [54]	Parent-reported sleep duration computed from bedtime and wake time	<37 weeks of gestation	6 months to 140 months	Approximately 350 at 140 months		x						Only estimated sleep duration from bedtime and wake-up time. Does not account for sleep onset latency of duration of night waking.
(Bulut et al., 2020) [55]	Parent report: Brief Infant Sleep Questionnaire	≤32 weeks of gestation without developmental delay, intraventricular haemorrhage grades 3 and 4, chronic disease, congenital anomalies, current teething or infections	12–31 months	40	↑ greater duration waking in PT group ↓ More frequent number of wakings in full-term group	x	x	x				Small sample size, relied on parent-report. Sample not representative of children born ≤32 weeks with neonatal morbidities. Included control group of term-born children
(Caravale et al., 2017) [56]	Parent report: Brief Infant Sleep Questionnaire and the Sleep Disturbance Scale for Children	<37 weeks of gestation Without cognitive, language, or motor delay or neurosensory impairment or genetic syndrome, or major congenital abnormalities	13–29 months	51	X both frequency of night waking and duration of nocturnal wakefulness	x	x	x			Greater sleep difficulties (nocturnal movement, restless during the night, breathing problems) compared with term group	Small sample size, relied on parent-report. Sample not representative of children born <37 weeks with developmental problems
(Gössel-Symank, Grimmer, Korte and Siegmund, 2004) [57]	Actigraphy	24–34 weeks of gestation, birthweight <1500 g. Twins, infants with serious infectious diseases e.g., HIV, genetic disorders, and cerebral palsy were excluded	20 months	17	Infants born PT had significantly less restful sleep (immobile time) and more restless sleep (moving time) than infants born at term	x	↓ Daily rest phase significantly shorter in infants born preterm					Small sample size, did not use an algorithm to calculate the duration of night waking
(Huang et al., 2014) [58]	Brief Infant Sleep Questionnaire	<37 weeks of gestation Without severe medical or congenital problems	6 months	68	↑ (number of night awakenings)	↑	↑		x		More time spent crying during the night respiratory symptoms; greater time spent breathing through their mouth and loud-noisy breathing and greater severity of sleep problems in PT versus term group. Of preterm infants, 81% had an apnoea–hypopnea index (AHI) >1 event/hour	Did not compare objective sleep data between the groups. Small sample size. Sample not representative of children born <37 weeks with medical problems
(Lupini et al., 2021) [59]	Parent report: Brief Infant Sleep Questionnaire	<37 weeks of gestation	0–36 months	417	x	x	x	x	x	Greater for parents of preterm children aged 12–36 months		Relied on parent-report
(Shimada et al., 1993) [60]	Parental observation of daily activities over 14 days every month until 12 months corrected age	<37 weeks of gestation without neonatal morbidities	From corrected age 35–39 weeks to 12 months	57		x	x				Delayed development of sleep-wake periodicity when referenced to post-natal weeks, but not post-conceptional weeks. No significant between-group difference in longest sustained sleep and longest sustained wake period at the same corrected ages	Relied on parental observation. Sample not representative of children born <37 weeks with neonatal morbidities
(Wolke et al., 1998) [61]	Parent-reported night waking problems	<37 weeks of gestation in two cohorts- South Finland (SF) and South Germany (SG)	5, 20, and 56 months follow up	305 (SF) 1703 (SG)	↓ at 5 months (SF) ↓ at 20 months (SG) No significant differences at 20 and 56 months in SF cohort and at 5 and 56 months in SG cohort							Relied on parental report of night waking
Sleep in the preschool period												
(Romeo et al., 2019) ^a^ [62]	Parent-reported: Sleep Disturbance Scale for Children	≤31 weeks of gestation with no history of major medical complications and above 10th percentile for GA-BW	3–6 years	146	Y (Combined difficulty initiating and maintaining sleep score)				Y (Combined difficulty initiating and maintaining sleep score)		Significantly higher scores for sleep-disordered breathing and sleep hyperhidrosis. No significant difference in parasomnias, disorders of excessive somnolence, and nonrestorative sleep scores	Sample not representative of children born ≤31 weeks without neonatal complications. Questionnaire does not differentiate between night waking and sleep duration. Did not include a term-born control group
(Durankus et al., 2020) [63]	Parent-report Children’s Sleep Habits Questionnaire (CSHQ)	<37 weeks of gestation, without diagnosed sleep disorder and without major congenital abnormality	4–6 years	137	X on CHSQ scale ↑ More parents in the PT group stated that their child would wake up during sleep	x		↑ More parents in the PT group stated that their child would wake up early	x		Significantly higher CSHQ total scores in the PT group. No significant difference in bedtime resistance, sleep anxiety, parasomnia, or sleep-disordered breathing subscales. Greater frequency of loud snoring. A greater proportion of children born PT had obstructive sleep apnoea symptoms (mouth breathing, hyponasal speech, loud snoring, difficulty in breathing). A greater proportion of children born very and moderately preterm had CHSQ scores above cut-off for sleep disorder compared with children born late preterm	Relied on parental reports of sleep problems

^a^ Control group comprised of community recruited children without developmental, mental or physical disabilities or long-term prescribed medication. x refers to no statistically significant difference between the preterm group and the term-born group. ↓ refers to statistically significant lower values for the preterm group ↑ refers to statistically significant higher values for the preterm group All studies refer to a term-born control group ≥ 37 weeks of gestation unless stated otherwise. PT refers to preterm.
